# How Does Courtroom Broadcasting Influence Public Confidence in Justice? The Mediation Effect of Vicarious Interpersonal Treatment

**DOI:** 10.3389/fpsyg.2020.01766

**Published:** 2020-07-29

**Authors:** Jian Xu, Cong Liu

**Affiliations:** ^1^School of Media and Communication, Shanghai Jiao Tong University, Shanghai, China; ^2^China Institute for Urban Governance, Shanghai Jiao Tong University, Shanghai, China; ^3^Institute of Cultural Innovation and Youth Development, Shanghai Jiao Tong University, Shanghai, China

**Keywords:** courtroom broadcasting, interpersonal treatment, procedural justice, public confidence, governance

## Abstract

The present study aimed to examine whether the applied practice of cameras in courtrooms plays a positive role in public confidence in legal authorities and how such impact may occur from the perspectives of the Group Value Model and the surrogacy effect. A convenience sample of 170 college students participated in this experiment. The control group read the written judgment of a civil case published online while the experimental group read the same judgment and watched the court trial video of that case. The overarching mediation model confirmed that there was a significant and indirect influence of video watching on confidence in justice in general. The key underlying mechanisms of this impact were the positive perception of the interpersonal treatment by the judge as well as the perceived fairness of the procedure. This study contributes to the currently limited research examining whether and, if so, how courtroom broadcasting promotes public trust through obtaining empirical evidence. It also expands the application of the Group Value Model to a vicarious interaction setting and provides evidence of the surrogacy effect in a civil legal context.

## Introduction

The emerging new media bring with them both opportunities and challenges for the justice system. Allowing cameras in courtrooms is considered a judicial reform in implementing the principles of an open trial and enhancing the credibility of justice, as well as the legitimacy of the government (Landry, 2008; Whiting, 2017; Jin, 2018). Over the past two decades, most countries have generally adopted an increasingly more open attitude toward the practice of courtroom broadcasting via both text and video. However, courtroom broadcasting is still in its explorative stage, and concerns remain regarding its ramifications.

Policies regarding cameras in courtrooms vary. Countries such as Germany, France, and Japan are among those that strictly prohibit any form of courtroom broadcasting to the outside world (Code of Criminal Procedure of France, 2006; Jin, 2018; Courts Constitution Act, 2019). In contrast, Brazil allows cameras in the courtroom during the entire trial process. Both Australia and the United Kingdom have made significant progress in opening the court trial process to the public. In 1999, the Federal Court of Australia became the first Australian court to broadcast live sound and images of judgment on the Internet (Federal Court of Australia, 2017; Mason and Stepniak, 2000). In Britain, filming has been authorized in the Supreme Court since it was created in 2009, and in some English and Welsh Court of Appeal cases since 2013 (The New York Times, 2020). Now, Australian and British courts have increasingly engaged with social media, allowing social media reports of court proceedings (BBC News, 2010; Federal Court of Australia, 2017). The United States has been actively experimental in the use of courtroom broadcasting. After two longitudinal pilot projects on video recording courtroom proceedings (conducted 1991–1994 and 2011–2015, respectively), nearly every state in the union has provisions to allow the media to use video cameras and microphones in courtrooms in some circumstances, and cameras are a routine sight at the trial court level in some states (Radio Television Digital News Association, 2020).

China has witnessed the most drastic reform in judiciary openness in the world. The interim measures of the Supreme People’s Court’s Publication of Judgment Documents in 2013 required all documents of the Supreme Law’s judgments, rulings, and decisions to be published online (except for special circumstances stipulated by law). Following that, in 2015, the Supreme People’s Court of China instructed all courts at different levels to broadcast all trials (except those disallowed by law) on its official website. It achieved the goal of having full access to and the coverage of 3521 courts across the country. By the end of 2019, over 6 million court trial videos had been broadcast on this website (about 150 daily), with a total visit volume of more than 21 billion people. This official website has become the largest live video website of government affairs in the world.

Despite the great global progress in judicial transparency in terms of broadcasting courtroom proceedings to the public, arguments about its ramifications and opportunities persist. In support of its positive impact, there is a consensus view that the live broadcast of court trials is conducive to regulating trial conduct, promoting judicial transparency and justice, and protecting the public’s right to know and the media’s freedom of expression. According to a longitudinal survey conducted from 2010 to 2014 in the United States by the Conference of Court Public Information Officers (CCPIO), more courts and judges use social media each year, more than half of court officials believe media should be allowed, and relatively fewer concerns about potential ethical problems, such as privacy, were expressed (CCPIO, 2014). Nevertheless, the opposing voices generally believe that there is the potential for physical and psychological interference, such as hindering of the trial order, heightening pressure on judges and juries, and affecting the parties’ words and deeds (Goldfarb, 1998). More than one-fifth of the judges and a similar percentage of the attorneys who participated in the Federal Court Centers’ pilot project expressed concerns that video recording might have some effect in decreasing public confidence in the judicial system (Federal Judicial Center, 2016). This is a particular concern given the impossibility of predicting and controlling how the media and the public will interpret such videos, a possible social and legal consequence of broadcasting court trials.

As such, it is important to examine through empirical studies whether the broadcasting of court trials on new media can indeed promote the public’s confidence in the judicial system, and, if so, what underlying mechanisms may maximize the public perception of justice.

## Literature Review

So far, direct evidence for a relationship between watching court trial videos and confidence in justice is negligible. However, two theoretical frameworks may shed light on the link between these two variables and provide clues as to the possible underlying mechanisms, namely, the surrogacy effect (Dai and Walther, 2018) and the Group Value Model (Lind and Tyler, 1988).

### The Surrogacy Effect: The Link Between Video Watching and Confidence in Justice

#### The Concept of the Surrogacy Effect

The surrogacy emerges in a situation that involves an audience (or observer), a surrogate, and a media figure (or target). Specifically, individuals can form impressions of a person by observing how he or she reacts to a surrogate—a person who is similar to the observer in some critical respects (Dai and Walther, 2018; Dai et al., 2019). Put simply, the surrogate’s direct interactions with the target will reflect how the target interacted with the observer (Walther, 2015). The surrogacy effect was developed based on the concept of parasocial interaction (PSI) in the traditional media era, which described how television audiences responded to media personae as if the communication was unmediated (Horton and Wohl, 1956). In this process, the media audience might be a spectator or a vicarious participant who alternatively and reciprocally takes on the roles of various actors (Horton and Strauss, 1957). Rubin et al. (1985) also found that the virtual experience of conversing with a celebrity might foster a pseudo-friendship for an individual, consisting of empathy, personal interest, and attributional confidence, even without the individual having met that celebrity in person.

In the new media era, an increasing number of politicians and government agents broadcast their opinions on the latest affairs, display their work in progress, or communicate with citizens on new media platforms (Park, 2010). Meanwhile, new media have made observable the interpersonal interactions between public figures and individuals representing a variety of social groups. Dai and Walther’s (2018) study indicated that positive interpersonal interaction, such as a target’s (e.g., a public figure) confirmatory reply to a surrogate (a layperson) on Twitter, increases the observer’s PSI with that target. To give a more specific example, a citizen may observe, in a live report or on social media, how a politician treats another citizen with whom he or she shares some similar aspects (e.g., social status or political affiliations), thus giving the citizen a projection of how they will be treated by the politician should they have any direct interpersonal interaction in the future. Consistent with this finding, a study revealed that candidates’ actual responses to voters’ comments on a social networking site increased the participants’ favorable attitudes toward the candidate (Utz, 2009).

#### Media Type and the Evaluation of Public Figures

While new media have garnered these public figures or government agents more public attention, little is known about the outcomes in terms of presentation management that the online showcase could afford them. Early studies provided some evidence that the use of different media types may influence the evaluation of local science authorities’ interpersonal fairness in different ways. The findings showed that newspaper consumption, but not TV exposure, promoted the expectation of respectful, polite, and dignified treatment from local scientific authorities (Besley et al., 2006). In the age of emerging new media, several studies have indicated that media presentations with interactive features garner more positive subsequent judgments from an audience. For example, Lee and Jang (2013) found that local politicians using a microblogging service (versus news articles) induced a stronger PSI, overall evaluation, and voting intention, although the effect only existed among less affiliative individuals. In line with this finding, an earlier study showed that compared to video or avatar communication modes, text-chat produced a lower level of intimacy and copresence, which is less likely to induce perceived interpersonal trust in a partner (Bente et al., 2008). Similarly, it was found that the conversational perception of a media message leads to higher ratings of trust, satisfaction, and commitment (Kelleher and Miller, 2006). These studies suggest that the more interactive the media are, or a public figure appears to be via those media, the more this fosters a sense of non-mediated interpersonal contact between the audience and the target, which in turn enhances subsequent evaluations.

Social media permanently display public figures’ interactions with individuals across multiple social groups and strata (Nonnecke and Preece, 2000; Marwick and Boyd, 2011). Similarly, broadcasting court trial videos online both record and publicly display the interpersonal interactions between legal authorities or officers and litigants, making these records readily available to all citizens. The observed interpersonal treatment of others by judges or court staff will, in the long run, contribute to the public’s evaluation and perceptions of justice in the legal system, even though the public audience has had no direct encounter with the legal authorities. Evidence from studies on the surrogacy effect and the effect of different media types suggests that watching how legal authorities/judges treat litigants on video may influence the formation of confidence in justice. Specifically, by observing the interpersonal interactions between judges and litigants via court trial video (as compared to reading judgment documents), respondents are exposed to more detailed cues about the interpersonal interactions in terms of the litigants’ presentation, the judges’ respectfulness, and so on, and are likely to become more confident in the fairness of any future treatment they may receive from the legal authorities. Hence, the following hypotheses are proposed:

H1. Watching the court trial video (X) increases confidence in justice (Y).
H2. Watching the court trial video (X) increases perceived interpersonal treatment (M1).
H3. Perceived interpersonal treatment (M1) increases confidence in justice (Y).
H4. The effect of watching the court trial video (X) on confidence in justice (Y) is mediated by interpersonal treatment (M1).

### A Group Value Model: Interpersonal Treatment, Procedural Justice, and Confidence in Justice

#### Interpersonal Treatment and Procedural Justice

Since Thibaut and Walker’s (1975) articulation of the psychological model of procedural fairness or procedural justice, process control has been widely accepted by researchers as a central factor that shapes people’s views about an authority’s fairness. This is especially true in dispute settings (Tyler, 1989). Process control, later called representation, refers to participants’ control over the presentation of evidence, which reflects the opportunities litigants have to present their problem or case to the authorities (Tyler, 1988). Based on Thibaut and Walker’s work, Lind and Tyler (1988) proposed a Group Value Model emphasizing people’s concerns about their relationships with third parties in evaluating procedural justice. Tyler’s (1988) empirical study found that among the various procedural justice criteria examined, representation, ethicality, and impartiality were the key factors significantly related to procedural justice, the evaluation of the legal authorities’ fairness, and even the expectation of the fairness of treatment in future encounters with the courts. Ethicality in the judicial context was defined as whether the courts followed the general principles of fair conduct, for example, whether the authorities were polite to the litigants, and whether they showed concern for the litigants’ rights. Impartiality or neutrality could be operationalized as unbiased and honest treatment and making efforts to be fair.

As proposed in the Group Value Model, interpersonal aspects such as trust, neutrality, and standing or voice have been given increasing emphasis in the development of procedural justice research regarding their influence on procedural justice judgments. Leventhal (1980) was among the first researchers who proposed the factors involving interpersonal interaction between the legal authorities and litigants that allow individuals to evaluate the facets of procedural justice, such as bias suppression, ethicality (i.e., procedures compatible with individuals’ moral values), and representation. As indicated in Tyler’s (1988) empirical study, the factors influencing an individual’s criteria for assessing procedural justice are those least linked to outcomes and most concerned with the interpersonal aspects of encounters with authorities: the legal authorities’ politeness, their concern for the litigants’ rights, honesty, and the litigants’ opportunities to present their viewpoint. In Tyler’s (1989) study testing the Group Value Model, it was suggested that besides the control issue (i.e., representation), the three non-control issues, that is, neutrality of the decision-making procedure, trust in the third party, and evidence about social standing are equally important in shaping the interpretation of one’s experience with the authorities, particularly in a dispute setting. As summarized in MacCoun’s (2005) review, two particularly important dimensions among the process aspects that shape procedural justice are voice (or representation, i.e., the ability to tell one’s story) and dignified, respectful treatment. In Tyler’s (2008) more recent study, four key procedural justice principles of the court experience that should be emphasized by legal authorities were summarized as voice (or representation), neutrality (or impartiality), respect (or ethicality or social standing), and trust. Similar findings have been replicated in policing, political, transactional, workplace, family, and university settings, in both cross-sectional and longitudinal studies (Bies and Shapiro, 1987; Tyler et al., 1996; Tyler, 2001; Gangl, 2003; Goodman-Delahunty, 2010; Mazerolle et al., 2013; Jonathan-Zamir et al., 2015; Pignata et al., 2016).

#### Procedural Justice and Confidence in Justice

The influence of procedural justice on litigant outcomes is threefold and depends on the litigant’s satisfaction with the outcome, evaluation of the legal authorities, and supportive behaviors to the authorities, respectively. Since the 1970s, researchers proposed and found empirical evidence that perceptions of the fairness of the dispute resolution process independently influence litigants’ satisfaction with dispute resolution decisions, notwithstanding the unfavorability or unfairness of those decisions (Thibaut and Walker, 1975; Lind, 1982; Walker and Lind, 1984; Landis and Goodstein, 1986; Lind and Tyler, 1988).

Studies have also supported that procedural justice influences litigants’ evaluations of the legal authorities and institutions responsible for settling disputes (Tyler and Folger, 1980; Tyler and Caine, 1981; Lind, 1982; Tyler, 1984, 2001; Tyler et al., 1986; Colquitt, 2001). According to the Group Value Model, the importance of procedural justice can be explained by the effect whereby it informs people about their social connection to group authorities in terms of group pride and respect within the group (Tyler et al., 1996).

Furthermore, procedural justice influences people’s reactions to judicial decisions. It is argued that procedural justice plays a key role in shaping the legitimacy that citizens grant to a government authority (Tyler, 2004). This legitimacy or support for the system is critical to the ability to govern effectively. Evidence from empirical studies supports the causal relationship between procedural justice and perceived legitimacy, which leads to policy acceptance (Tyler and Lind, 1992; Tyler, 2004; MacCoun, 2005). In addition, relational procedural justice judgments influence individuals’ group-oriented behaviors, including acceptance of the court’s decision, compliance with the law, and support for the government (Tyler et al., 1996, 2015; Tyler, 2004; Skogan, 2006; Dai et al., 2011). Scholars have also long noted the positive social benefits of procedural justice in promoting social harmony and cooperation in the face of divergent interests and inevitable scarcity (MacCoun, 2005). The positive effects of procedural justice have also been found in legal, industrial, political, and interpersonal settings (Tyler et al., 1996; Blader and Tyler, 2003; Brienza and Bobocel, 2017).

Confidence in the legal system could be a core underlying mechanism between procedural justice and supportive behaviors toward legal authorities. Sunshine and Tyler’s (2003) study found that legitimacy, in which the public’s confidence in authorities or legal actors plays a significant role, mediates the link between procedural justice and the public’s empowerment of and cooperation with police and their compliance with the law. Tyler’s (1989) study also revealed a strong intercorrelation between confidence in legal authorities and procedural justice. Similarly, it was found that a procedure’s fairness enhances people’s belief that they will receive a fair outcome and be treated justly if they go to court in the future (Tyler, 2001). Based on the existing literature, it appears that a positive evaluation of interpersonal treatment fairness in court enhances procedural justice judgments, which will, in turn, promote confidence in justice. Therefore, we hypothesized the following:

H5. Perceived interpersonal treatment (M1) increases perceived procedural justice (M2).
H6. Perceived procedural justice (M2) increases confidence in justice (Y).
H7. The effect of perceived interpersonal treatment (M1) on confidence in justice (Y) is mediated by perceived procedural justice (M2).

From the perspectives of the surrogacy effect and the Group Value Model, and taking H1–H5 together, we can draw a link sequentially from watching court trial video (X) to interpersonal treatment (M1), to procedural justice (M2), and to confidence in justice (Y). Therefore, we hypothesized that:

H8: There is a chain mediation between watching court trial video (X) and confidence in justice (Y) through perceived interpersonal treatment (M1) and perceived procedural justice (M2).

### The Influence of Personal Experiences

The fairness heuristic theory indicates that people appear to make greater use of fairness judgments when they experience uncertainty (Lind and Van den Bos, 2002). People who have experienced uncertainty (i.e., a prior victimization experience) tend to rely more on procedural fairness than their counterparts when judging the trustworthiness of the police (Wolfe et al., 2016). In the judicial context as discussed in the present study, the participants may have had personal experience with legal authorities themselves or have friends or relatives working as legal authorities. Experiencing such direct contact with legal authorities instead of vicariously could reduce uncertainty and, therefore, undermine the surrogacy effect in relation to the court trial video. Hence, it is plausible that for participants who have had no previous contact with legal authorities, watching video facilitates their positive interpersonal treatment perceptions, while for those who had such experience or contact, the video’s effect could be weaker. Therefore, we proposed the following hypothesis:

H9: Direct personal contact with legal authorities moderates the effect of video watching on interpersonal treatment perception. Specifically, video watching will increase the interpersonal treatment perception of those who have had no contact with legal authorities compared to those who have had such experience.

## The Present Study

Despite the vast move toward judicial transparency promotion via new media, we know of no published research examining how courtroom broadcasting might influence the fairness of legal authorities and public trust in the legal system. As reviewed above, although studies have examined how the use of different media types may influence the authority evaluations by an audience, no content control was attempted across the media. Also, there is a dearth of studies exploring the underlying mechanisms of the media’s effect on public trust. The Group Value Model was developed to explain how interpersonal treatment influences authority evaluations, but it is mostly applied to the context of direct encounters with authorities or legal actors and not the setting of vicarious experience through new media, which are more accessible to the majority of citizens.

**FIGURE 1:**
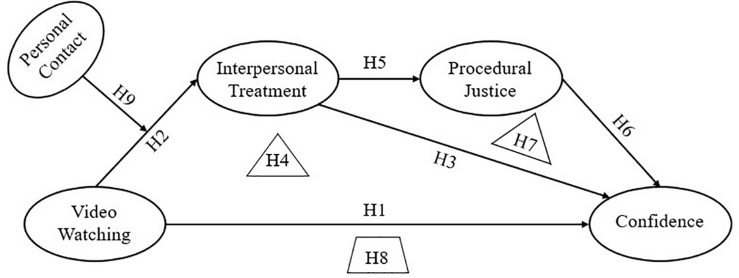
Conceptual framework.

As such, the present study aimed to discern whether the practice of placing cameras in courtrooms exerts a positive influence on the public’s confidence in legal authorities, and how the impact occurs in light of the Group Value Model and the surrogacy effect. To integrate the study’s hypotheses into one overarching model, a conceptual framework was posited as shown in Figure 1.

## Materials and Methods

### Participants

A total of 170 college students from China participated in the experiment^1^. Each participant was given 40 yuan (approximately 5.80 USD) as an incentive for participation. Among them, 44.1% were male. The participants were aged between 18 and 31 years, with an average age of 22.8 years (*SD* = 2.21), and composed of 17.0% undergraduate, 56.5% masters, and 26.5% Ph.D. students. Only 2.4% were majoring in law. The majority of participants (62.4%) believed they were from a family with an average socioeconomic status. More than one third (35.3%) had personal contact with legal authorities (i.e., had personal experience with the police or legal authorities or had contact with someone working in the legal system). The participants’ demographic characteristics are shown in Table 1.

**TABLE 1:** Demographic characteristics of the participants.

	Frequencies	Percentage
**Gender**		
Male	75	44.1
Female	95	55.9
**Age**	**(Min = 18; Max = 31; Mean = 22.77; *SD* = 2.21)**	
18–25	149	87.6
Above 25	21	12.4
**Education**		
Undergraduate	29	17
Master	96	56.5
Ph.D.	45	26.5
**Socio-economic status**		
Average	106	62.4
Middle class	60	35.3
High status	4	2.4

### Experimental Design

#### Case Selection

A real-life civil case considered to be typical of many others and that received widespread public attention in 2008 was selected as the stimulus material. The selected case outlined the plaintiff Wang Fei’s dispute over the defendant Zhang Leyi’s infringement of Wang’s reputation and privacy rights. Wang and his wife Jiang were a young couple living in Beijing. Wang divorced Jiang because of his extramarital affair. After learning the truth about the divorce, Jiang became depressed and committed suicide. On her personal blog, she posted a narrative of her journey from her divorce dispute with her husband to her suicidal decision. Zhang, Jiang’s former boyfriend during college, posted the incident and his comments on a website in memory of Jiang’s death and disclosed Wang’s personal information. Wang sued Zhang for impugning his reputation and infringing his right to privacy. The local court held that Wang’s extramarital affair not only violated the law but also deviated from social and moral standards. At the same time, the court supported Wang’s claim that Zhang’s behavior violated Wang’s rights to privacy and damaged his reputation, and that Zhang should stop the infringement, apologize, and compensate for the corresponding economic and psychological losses.

#### Experimental Conditions

The experiment examined the unique contribution that courtroom videos may add to public confidence in the justice of legal authorities beyond other more accessible and less controversial forms of information about court proceedings, for example, the written judgment. There were two experimental conditions (control: written judgment; experimental: written judgment and court trial video of the case). The written judgment was the full version of the local court’s civil judgment released online, including a brief introduction about the relationships among the persons involved. The video was an edited version of a legal program reporting this case, which contained the complete story as well as many clips of the court trial, including the trial preparation, the statement of parties, the presentation of evidence, both parties’ closing arguments, and the case review and judgment announcement. The video clips involved the judge, court staff, litigants, and their attorneys. The length of the video was about 27 min.

### Questionnaires

#### Interpersonal Treatment

Based on the Group Value Model (Tyler, 1989, 2008), five items measuring respect, neutrality, trust, and voice were adapted from Tyler’s (1988) study: “How polite were authorities to the litigants?” (respect), “How much concern did the authorities show for the litigants’ rights?” (trust), “How much improperness or dishonesty did the authorities show?” (reverse coded; trust), “Was the treatment or outcome influenced by the litigants’ race, sex, age, nationality, or some other personal characteristic?” (neutrality), and “How much opportunity did both litigants have to present their problem or case to the authorities before the decisions were made?” (voice). Responses were made on a five-point scale (ranging from 1 = “very little” to 5 = “very much”). Cronbach’s alpha in this study was 0.73.

#### Procedural Justice

The measurement of procedural justice was adapted from Tyler’s (1988, 1989) questionnaire that assessed judgments about the fairness of the procedures, composed of two questions, “How fair were the procedures used by the authorities?” and “How fairly were the litigants treated?” Responses were made on a 5-point scale (ranging from 1 = “very unfair” to 5 = “very fair”). Cronbach’s alpha in this study was 0.73.

#### Confidence in the Justice of Legal Authorities

A key assumption underlying the concept of confidence in legal authorities is that people tend to predict from the past to the future, and to generalize from part to whole, as mentioned above (Tyler, 1989). It was of interest in this study to examine the surrogacy effect, that is, the projection of an observation of another citizen’s experience with the authority onto the expectations of one’s own future encounters. Therefore, we adapted items regarding the participants’ expectations of the legal authorities to measure their confidence in justice. Three items were adapted from Tyler’s (2006) questionnaire for interviewing citizens about their experience with courts: “How fairly do you think you will be treated by the courts if you are to deal with them in the future?”, “How satisfied do you think you will be about the outcomes you will receive from the courts in the future?”, and “How fair do you think the outcomes you will receive from the courts in the future will be?” Responses were made on a five-point scale (from 1 = “not at all” to 5 = “very much”). Cronbach’s alpha in this study was 0.81.

#### Demographic Variables

The demographic variables, as displayed in Table 1, included age, gender, education (undergraduate/masters/Ph.D.), major in law (yes/no), socioeconomic status (average/middle class/high), and personal contact with legal authorities (yes/no).

### Pilot Study

The purpose of the pilot study was to confirm participants’ comprehension of the case and the relationships among the involved persons from reading the written judgment and viewing the edited court trial video. A total of 25 students participated in the pilot study. They were assigned to either the control condition (12 participants) or the experimental condition (13 participants), as described in previous sections. As a manipulation check, the participants were interviewed after reading or viewing and asked to answer a few simple questions such as “What was the defendant’s surname?” and “What was the relationship between Jiang and Zhang?” The pilot study confirmed that all participants in both experimental conditions were clear about the case and the relationships among the persons. Minor changes/supplements were made according to the participants’ suggestions (for example, a brief identity/relationship list of the involved persons was added at the beginning of the judgment document).

### Procedures

Data collection for the main experiment took place over approximately 3 months. Before the experiment was started, the participants were introduced to the study aims, procedures, and rules. Informed consent was obtained from each participant. Participants were then randomly assigned to either the control group (*n* = 70) or the experimental group (*n* = 100). The participants in the control group were asked to read the written document, while the participants in the experimental group were asked to read the written judgment and watch the court trial video. To counterbalance the order effect, half the participants read the written judgment first, and the other half watched the video first. The participants were required to wear a headset while watching the video. Both groups completed the same questionnaires after the experiment. The control group spent an average of 20 min completing the experiment, while the experimental group spent an average of 40 min. Two research assistants supervised the experiment and a maximum of six participants simultaneously participated in the experiment. Discussion was not allowed until they had completely finished the experiment and questionnaire. All participants confirmed that it was the first time they had learned about the presented case.

## Results

### Descriptive Statistics and Correlations

The means and standard deviations (*SD*s) for interpersonal treatment, procedural justice, and confidence in justice scores according to experimental group are shown in Table 2. The mean scores for interpersonal treatment were 22.87 (*SD* = 2.16) and 21.90 (*SD* = 2.59) for the experimental and control groups, respectively (*F* = 7.05, *p* < 0.01; Cohen’s *d* = 0.41). The mean scores for procedural justice were 4.41 (*SD* = 0.61) and 4.21 (*SD* = 0.57) for the experimental and control groups, respectively (*F* = 4.80, *p* < 0.05; Cohen’s *d* = 0.34). The mean scores for confidence were 3.77 (*SD* = 0.55) and 3.69 (*SD* = 0.54) for the experimental and control groups, respectively (*F* = 1.07, *p* > 0.05; Cohen’s *d* = 0.15).

**TABLE 2:** Descriptive statistics and intercorrelations of key variables.

	Video Watching (1)	Mean	*SD*	Cohen’s *d*	*F*	(1)	(2)	(3)	(4)
						**1**			
Interpersonal treatment (2)	Experiment	22.87	2.16	0.41	7.05**	0.20**	1		
	Control	21.90	2.59			(0.009)			
Procedural justice (3)	Experiment	4.41	0.61	0.34	4.80*	0.17*	0.65***	1	
	Control	4.21	0.57			(0.030)	(0.000)		
Confidence (4)	Experiment	3.77	0.55	0.15	1.07	0.08	0.40***	0.44***	1
	Control	3.69	0.54			(0.302)	(0.000)	(0.000)	

*p-*values are in parentheses after the correlation coefficients. *p < 0.05, **p < 0.01, ***p < 0.001.

The grouping variable “video watching” was created based on the two experimental conditions (0 = control condition, 1 = experimental condition). A Pearson correlation analysis showed that video watching was positively and significantly correlated with interpersonal treatment (*r* = 0.20, *p* < 0.01) and procedural justice (*r* = 0.17, *p* < 0.05) but not confidence. Further, interpersonal treatment was positively and significantly correlated with procedural justice (*r* = 0.65, *p* < 0.001) and confidence (*r* = 0.40, *p* < 0.001). Finally, procedural justice was positively and significantly correlated to confidence (*r* = 0.44, *p* < 0.001).

### Test of the Chain Mediation Model

The chain mediation model incorporating H1 to H8 as shown in Figure 1 was tested with Model 6 of the SPSS PROCESS MACRO (Hayes, 2013), in which Y = confidence, X = video watching, M1 = interpersonal treatment, M2 = procedural justice. All analyses computed a 95% bias-corrected confidence interval (CI) with 5000 bootstrap resamples. Two samples with missing data were deleted from this analysis.

Results of the chain mediation model testing are shown in Table 3^2^. In the first regression predicting interpersonal treatment, the effect of video watching was positive and significant (*b* = 0.96, *SE* = 0.37, *p* < 0.05) and H2 was supported. In the second regression predicting procedural justice, the effect of interpersonal treatment was positive and significant (*b* = 0.16, *SE* = 0.02, *p* < 0.001), while the effect of video watching was not significant (*b* = 0.05, *SE* = 0.07, *p* > 0.05), and H5 was supported. In the third regression predicting confidence, the effects of interpersonal treatment (*b* = 0.05, *SE* = 0.02, *p* < 0.05) and procedural justice (*b* = 0.28, *SE* = 0.08, *p* < 0.01) were both positive and significant (thus, H3 and H6 were supported), while the effect of video watching was not significant (*b* = -0.02, *SE* = 0.08, *p* > 0.05).

**TABLE 3:** Mediation model testing the effect of video on confidence through interpersonal treatment and procedural justice.

	Outcome 1: Interpersonal treatment				Outcome 2: Procedural justice				Outcome 3: Confidence			
	*b*	*SE*	*t*	*p*	*b*	*SE*	*t*	*p*	*b*	*SE*	*t*	*p*
Video watching (1 = exp, 0 = ctrl)	0.96**	0.37	2.62	0.009	0.05	0.07	0.66	0.511	–0.02	0.08	–0.21	0.830
Interpersonal treatment					0.16***	0.02	10.64	0.000	0.05*	0.02	2.34	0.020
Procedural justice									0.28**	0.08	3.34	0.001
*R*^2^(*F*)	0.04(6.86)**				0.42(60.67)***				0.22(15.42)***			

*N* = 168. *p < 0.05, **p < 0.01, ***p < 0.001.

Results testing the direct, indirect, and total effects of video watching on confidence showed that there was a significant total effect (*b* = 0.10, Boot *SE* = 0.04, 95% Boot CI [0.027,0.206]), no significant direct effect (*b* = −0.02, Boot *SE* = 0.08, 95% Boot CI [−0.171,0.138]; H1 was not supported), and two significant indirect effects. One of the significant indirect effect of video watching on confidence was through interpersonal treatment (*b* = 0.05, Boot *SE* = 0.03, 95% Boot CI [0.008,0.126]) and H4 was supported. The other significant indirect effect was through interpersonal treatment and procedural justice (i.e., a chain mediation model from video watching to interpersonal treatment to procedural justice and to confidence; *b* = 0.04, Boot *SE* = 0.02, 95% Boot CI [0.011,0.097]), which indicated that procedural justice was a significant mediator between interpersonal treatment and confidence^3^ and thus H7 and H8 were supported.

**FIGURE 2:**
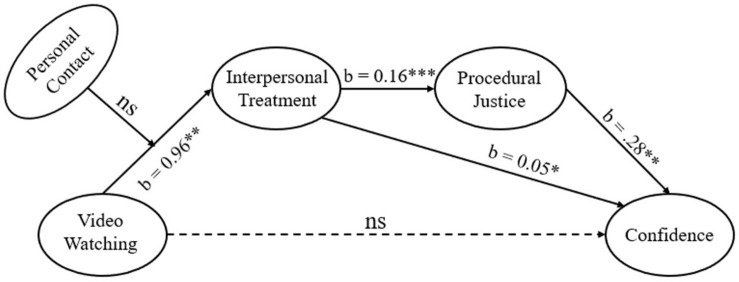
Chain mediation model illustrating the link between video watching and confidence.

To test the moderation effect of personal contact on the link between video watching and interpersonal treatment, a model was built using Model 7 of the SPSS PROCESS MACRO (Hayes, 2013). All analyses computed 95% bias-corrected CIs with 5000 bootstrap resamples. The results showed that personal contact had no significant moderation effect on the link between video watching and interpersonal treatment (*b* = 1.37, *SE* = 0.76, *p* = 0.075) and thus H9 was not supported. The overarching chain mediation model is shown in Figure 2.

## Discussion

Many countries are on the path to practicing judicial transparency, although the use of new media to do so remains ambiguous and controversial. We know of no existing research that examines the consequences of the controversial practice of cameras in courtrooms and broadcasting court proceedings on new media in terms of public confidence in the justice of the legal system. The empirical evidence from the present study gives new insights into the answers to the following questions: (a) Does exposing the public to court trial videos lead to greater confidence in justice; (b) If so, what are the underlying mechanisms; and (c) What is the takeaway advice for the legal authorities and policymakers?

In brief, we found that there is no direct effect of video watching on confidence in justice. However, video watching has two mediated paths in which it exerts influence on confidence: one is through interpersonal treatment, and the other is through interpersonal treatment and procedural justice.

### Effects of Video Watching on Confidence in Justice

The main research question in this study was whether exposing the public to court trial videos results in higher public confidence in justice, and more importantly, how this might work. There was no direct effect of video watching on confidence. The only significant direct effect of video watching was on interpersonal treatment. Although this result failed to support our first hypothesis, Zucker’s (1986) trust production theory could provide an alternative explanation. Confidence in the justice of legal authorities can be decomposed into two aspects, namely, institution-based trust and process-based trust. People obtain confidence in justice at the institutional level, where legitimacy, as well as other previously formed evaluations of the legal authorities, are key factors. However, people also gain confidence in justice at the process-based level, which is developed through interaction(s) or encounter(s) with the trustee. Both mechanisms of trust production connote that the formation of confidence in justice cannot be developed through a brief, single exposure to court trial procedures, let alone vicariously. In contrast, the cues relating to interpersonal treatment by the judge are very likely to be perceived by the audience even if there is only one short-term exposure. Several studies support the assertion that a single, mediated encounter can enhance parasocial relationships and positive impressions with the person on media as long as additional social cues are presented (Tidwell and Walther, 2002; Tanis and Postmes, 2003; Lee and Jang, 2013). These findings may explain why watching one video clip of a court trial may increase the perception of the interpersonal treatment of the judge but not confidence in justice, as found in the present study.

The non-significant direct effect of video watching on confidence as well as the significant mediation effects also suggest that simply increasing the amount of court trial broadcasting may not be an efficient way of promoting public confidence in justice. It was demonstrated that the quality of court trials in terms of how the judges and other court staff work and treat the litigants is important. It is unclear, based on this study, whether increased broadcasting of court trials of greater quality in this regard will eventually promote public confidence in justice. However, we have at least shed light on the directions that the legal system and relevant sectors could make to help realize the goal of higher public confidence.

### Interpersonal Cues in Courtroom Broadcasting

One of the objectives of the present study was to extract the core elements of the court trial videos that could most effectively promote people’s impressions and evaluations on the legal procedures and justice. The most noteworthy finding in the present study is the mediating role of interpersonal treatment. That is to say, audiences of court trial videos who perceive a judge’s positive interpersonal treatment toward a litigant tend to be more confident about receiving fair treatment and outcomes in their own future encounters with the legal authorities. This is in line with findings in previous studies showing that a mediated communication with higher social presence (e.g., operationalized in terms of sociability, warmth, and sensitivity) of the person depicted in the media will lead to a more positive impression on the audience (Short et al., 1976; Lombard and Ditton, 1997). Courtroom videos create a higher sense of the social presence of the judge compared to written judgments. Therefore, courtroom videos induce parasocial interactions between the audience and the judge, and the quality of such interactions can shape their expectancies toward legal authorities. Most previous studies on the surrogacy effect or parasocial interactions have been conducted in a text-based context (e.g., microblogging or news articles) and among celebrities or politicians (Lee and Jang, 2013; Dai and Walther, 2018). This study is novel in broadening the application of the findings from the surrogacy effect to the video setting and in the legal context, among lesser-known judges in particular.

Adding to Tyler’s (1989) proposition that people tend to predict from their past and limited experience to the future and general situations, the results from the present study further suggest that people tend to transfer others’ experiences to their own, even if the experience was observed in a mediated manner. Coincidently, evidence in support of both the Group Value Model and the surrogacy effect share a common inherent mechanism: social identification (Tyler, 1989; Tyler and Lind, 1992; Dai and Walther, 2018). However, the distinction is that the former emphasizes the social identification obtained from the authorities, while the latter emphasizes that with the surrogate. Specifically, Tyler (1989) argued that people care about their long-term membership of their group and its authorities or institutions. Therefore, they need information regarding their group standing as well as their fair benefits from the group, which are linked to the interpersonal aspects of interaction with authorities. The surrogacy effect emphasizes that participants’ identification with a surrogate who has direct interaction with a public figure is central. Taken together, regardless of whether the identification was between the observer and the surrogate or between the individual and the authority, the inherent meaning of identification is equality among human beings and their sense of belonging to the group (be that a community or authority).

Personal contact was found not to moderate the link between video watching and perceived interpersonal treatment, contradicting our hypothesis in this regard. Although existing studies indicate that the anxiety and psychological discomfort caused by uncertainty in a legal setting can be undermined by direct experience or personal contact with the authorities (Lind and Van den Bos, 2002; Wolfe et al., 2016), such negative feelings may be too weak or negligible to make a sufficient difference through vicarious observation as tested here. This explains why the moderation effect of personal contact was not found in this study.

### Interpersonal Antecedents of Procedural Justice Based on Vicarious Experience

Findings also suggest that procedural justice is a key mediator between interpersonal treatment and confidence in justice. Procedural justice has long been noted as a key factor that enhances individuals’ favorability to the outcome of distribution by an authority or institution and their evaluations, such as their trust in and positive affect toward, and their perceived legitimacy of the authorities or institutions. It also induces supportive intentions such as compliance, cooperative behaviors, and voting (Tyler, 1989; Colquitt, 2001; Skogan, 2006; Dai et al., 2011). Such findings have been replicated in multiple settings. Among the antecedents of procedural justice, interpersonal aspects have been given an increasing emphasis by scholars and practical actors, as suggested by the Group Value Model. In line with this theory, the present study also suggested that a judge’s interpersonal treatment is positively related to the perceptions of procedural justice by the audiences. However, most existing studies assessing participants’ relational treatment perceptions have been based on participants’ direct interactions with an authority or institution (Bies and Shapiro, 1987; Lind and Tyler, 1988; Tyler, 1988, 1989, 2001; Tyler et al., 1996; Gangl, 2003; Mazerolle et al., 2013; Jonathan-Zamir et al., 2015; Pignata et al., 2016). Meanwhile, very few studies have investigated or discussed how the interpersonal cues observed in a vicarious video experience could impact on perceived procedural justice, which, in turn, increases the participants’ confidence in justice. The findings from the present study suggest that the Group Value Model can be generalized to a more intangible setting, that is, the observation of others’ experiences through media.

Two theories provide a more in-depth understanding of the relationships among interpersonal treatment, procedural justice, and confidence, namely, social identification theory and the fairness heuristic theory. Social identification is a key mechanism that explains why interpersonal treatment, such as the right to present oneself and receive dignified treatment, is important in the evaluation of procedural justice. It is argued that people obtain information about their group standing as well as whether their rights are respected based on the interpersonal treatment they receive during social interactions. Polite and respectful treatment indicates high status in the group and, similarly, respect for their rights (Tyler, 1989; Tyler et al., 1996). The fairness heuristic theory proposes that knowledge about processes and outcomes both serve to reduce uncertainty about others’ motives in a substitutive manner. In other words, when information is lacking about an outcome, procedural justice serves as a heuristic substitute (Van den Bos, 2001; Lind and Van den Bos, 2002; Van den Bos and Lind, 2002; MacCoun, 2005). This could explain why a procedure’s perceived fairness was found to be positively related to one’s belief in receiving fair treatment and outcomes in future encounters with an authority.

### Implications and Limitations

This study could be among the first empirical studies to confirm that broadcasting court trials online can be very meaningful in enhancing public trust in the legal system if managed appropriately. We have shown that videos make a unique contribution to this process, adding to that of written judgments. Furthermore, this study suggests that judges’ interpersonal treatment cues can be clearly perceived by the audience in a mediated communication, even when they are very subtle and not in any circumstances an intended highlight of the video. There is scope for legal authorities, judges, and court staff, in particular, to enhance their public impressions by paying more attention to their specific conduct in court, especially in how much respectfulness they show, how unbiased they appear to be, how politely they treat the litigants, and how much opportunity they give the litigants to make their voices heard. Interpersonal procedural justice could be equally important to the public as the fairness of trial’s judicial procedures. Last but not least, there are concerns by scholars that authorities can utilize the appearance of fair procedures (via factors such as dignity, respect, and voice) as an inexpensive way to distract citizens from substantively unfair or biased outcomes (MacCoun, 2005), or that legal proceedings could become a show for the legal authorities to earn public trust.

There are some limitations to the present study. First, as increasingly more written judgments, courtroom photos, and video clips of court trials are broadcast on social media, it is worth exploring whether exposure to such fragmented information also has similar effects. Second, a civil case was used in this experiment, but criminal cases, which usually garner more public attention, should be explored in future studies and comparisons between these two case types could be made. Third, this study recruited a convenience sample among college students. The sample should be expanded to larger social strata in future studies.

## Conclusion

This study builds on the existing limited studies in this field by obtaining empirical evidence relating to how courtroom broadcasting may promote public trust in justice. It confirmed the link between courtroom broadcasting and public trust in justice by highlighting the importance of positive interpersonal cues displayed by the judges and the court staff in proceedings. This study contributes theoretically by expanding the application of the Group Value Model to a vicarious interaction setting and adding evidence of the surrogacy effect in a civil legal context.

## Data Availability Statement

The datasets generated for this study are available on request to the corresponding author.

## Ethics Statement

The studies involving human participants were reviewed and approved by the Shanghai Jiao Tong University. The patients/participants provided their written informed consent to participate in this study.

## Author Contributions

Both authors listed have made a substantial, direct and intellectual contribution to the work, and approved it for publication.

## Conflict of Interest

The authors declare that the research was conducted in the absence of any commercial or financial relationships that could be construed as a potential conflict of interest.

## Footnotes

1 To maintain an adequate power of 80% to detect differences between groups, 30 participants per cell was suggested to provide a medium to large effect size (Cohen, 1988). Lower expected effect sizes require more participants to maintain adequate power (Aron and Aron, 1999). For regression analysis, Green (1991) suggested that *N* > 50 + 8*m* (*m* is the number of IVs, which is three in the present study). According to previous studies, we expected small to medium effect sizes in the present study (Lind and Tyler, 1988; Dai and Walther, 2018). Based on the above calculations, 170 participants were considered adequate to maintain a power larger than 80% for both the ANOVAs and regression analysis.

2 Results of mediation-model testing after controlling for the effects of the demographic variables are provided in Supplementary Table S1.

3 No significant or direct effect was found between video watching (X) on confidence (Y) in the mediation model in this study. Most contemporary analysts believe that the direct effect of the predictor variable on the outcome variable is not required (Kenny et al., 1998; Kenny, 2018), and it could be termed “indirect-only mediation” as suggested by some researchers (Zhao et al., 2010).
