# Supplementary material for: How Does Courtroom Broadcasting Influence Public Confidence in Justice? The Mediation Effect of Vicarious Interpersonal Treatment
Source: Front Psychol. 2020 Jul 29;11:1766. doi: 10.3389/fpsyg.2020.01766 (PMC7403225; doi:10.3389/fpsyg.2020.01766)
Supplement: Supplementary file 1 [file Table_1.docx]

**Supplementary Material**

Supplementary Table S1

*Mediation Model Testing the Effect of Video on Confidence through Interpersonal Treatment and Procedural Justice with Demographic Variables Controlled*

|  | Outcome 1:  Interpersonal Treatment | | | | Outcome 2:  Procedural Justice | | | | Outcome 3:  Confidence | | | |
| --- | --- | --- | --- | --- | --- | --- | --- | --- | --- | --- | --- | --- |
|  | *b* | *SE* | *t* | *p* | *b* | *SE* | *t* | *p* | *b* | *SE* | *t* | *p* |
| *Covariates* | | | | | | | | | | | | |
| Gender | .58 | .38 | 1.54 | .124 | .03 | .07 | .45 | .651 | .01 | .08 | .10 | .918 |
| Age | -.03 | .11 | -.24 | .813 | -.03 | .02 | -1.17 | .241 | .02 | .02 | 1.05 | .294 |
| Education | -.05 | .36 | -.14 | .888 | .01 | .07 | .13 | .895 | -.01 | .08 | -.15 | .879 |
| Major (Law) | -.40 | 1.20 | -.34 | .736 | -.22 | .23 | -.96 | .341 | .03 | .25 | .11 | .910 |
| SES | .09 | .34 | .27 | .786 | .05 | .07 | .81 | .420 | .00 | .07 | .02 | .985 |
| *Predictors* | | | | | | | | | | | | |
| Video watching  (1 = exp,  0 = ctrl) | .90* | .37 | 2.44 | .016 | .05 | .07 | .65 | .514 | -.02 | .08 | -.27 | .784 |
| Interpersonal  treatment |  |  |  |  | .16*** | .02 | 10.34 | .000 | .05* | .02 | 2.26 | .025 |
| Procedural  justice |  |  |  |  |  |  |  |  | .29*** | .08 | 3.43 | .000 |
| *R*^2^(F) | .06(1.65) | | | | .44(17.88)*** | | | | .23(5.86)*** | | | |

*Note.* *N* = 168.
